# B-Cell Lymphoma Producing IgM Anti-B Antibody: A Case Report

**DOI:** 10.3389/fmed.2022.904296

**Published:** 2022-05-16

**Authors:** Feiyu Jiang, Tiejun Song, Yingjian Wang, Zhiwei Liu

**Affiliations:** Department of Blood Transfusion, Sir Run Run Shaw Hospital, Zhejiang University School of Medicine, Hangzhou, China

**Keywords:** B-cell lymphoma, ABO blood group discrepancy, case report, serology, genotyping

## Abstract

ABO blood group system is the most important blood group system in transfusion and transplantation medicine. Diffuse large B-cell lymphoma (DLBCL) is the most common type of non-Hodgkin lymphomas (NHLs) worldwide. There have been some studies that lymphoma could affect ABO blood group system and thus affect blood transfusion strategy. However, the mechanisms lymphoma affecting ABO blood group system have not been fully elucidated so far. Here, we report a case of a patient who was a 72-year-old Chinese man came to our hospital for medical advice because of cervical lymphadenophathy. The patient was subsequently diagnosed with diffuse large B-cell lymphoma by lymph-node biopsy. His ABO blood group was initially typed as B on November 7, 2020. He was transfusing B type leukocyte poor RBCs (LPR) before we found the patient’s ABO blood group discrepancy on December 2, 2020 by forward and reverse typing methods, which the discrepancy was verified by genotyping. The patient began to transfuse O type washed RBCs (WRBC) since then. Compared to transfuse B type leukocyte poor RBCs (LPR), the efficiency of transfusing O type washed RBCs (WRBC) was better. Although hemoglobin level did not greatly improve, indirect bilirubin level evidently decreased. Furthermore, we found B-cell lymphoma affected blood transfusion strategy by producing IgM anti-B antibody in this case. Clinicians should need to be aware of the effect of B-cell lymphoma on blood transfusion strategy.

## Introduction

**FIGURE 1:**
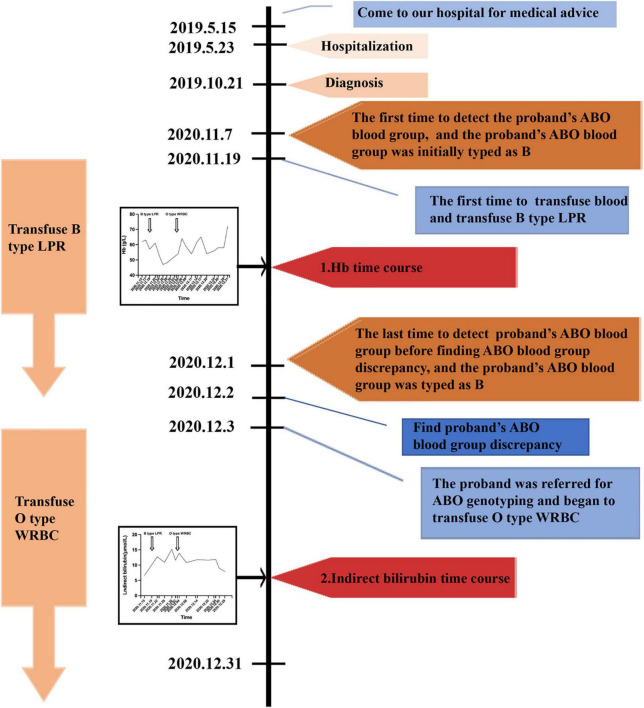
A timeline of the patient’s medical history and course of care. Hb, hemoglobin; LPR, leukocyte poor red blood cell; WRBC, washed red blood cell.

The ABO blood group system is the first blood group system discovered in humans and the most important blood group system in transfusion and transplantation medicine (1–4). The distribution and diversity of ABO blood group were crucial for determining novel ABO blood group and offering useful information in blood transfusion. Non-Hodgkin lymphomas (NHLs) are malignant disorders originating in immune cells and manifest predominantly as lymphadenopathy or solid tumors. According to the latest World Health Organization classification, NHLs are classified into more than 50 different subtypes (5). Diffuse large B-cell lymphoma (DLBCL) is the most common type of NHLs worldwide, representing approximately 30–40% of all cases in different geographic regions (6, 7). About 50–70% of cases of DLBCL express surface or cytoplasmic immunoglobulin, most often IgM followed by IgG and IgA (8). Erythrocyte autoantibodies are an uncommon complication of NHLs. A study showed that lymphoma could produce anti-B1 cold agglutinin, resulting in discrepancy of forward and reverse typing (9). But the deeper relationships between ABO blood group typing and lymphoma have not been elucidated. Here, we report a case of B-cell lymphoma producing IgM anti-B antibody. In addition, we found a rare O07 allele and the efficiency of transfusing O type washed RBCs (WRBC) for the patient was better compared with transfusing B type leukocyte poor RBCs (LPR). A timeline of the patient’s medical history and course of care is depicted in Figure 1. This case report was prepared following the CARE Guidelines (10).

## Case Presentation

A 72-year-old Chinese man came to our hospital for medical advice because of cervical lymphadenophathy on May 15, 2019. The patient had reported no significant past illness and had no history of blood transfusion. He was firstly admitted to our hospital on May 23, 2019 and was subsequently diagnosed with diffuse large B-cell lymphoma by lymph-node biopsy on October 21, 2019. The patient had tried a range of treatment regimens to unsatisfactory effect before being diagnosed with diffuse large B-cell lymphoma. He had been receiving a total of four RCHOP [addition of rituximab (R) to CHOP (cyclophosphamide, doxorubicin, vincristine, and prednisone)] immuno-chemotherapy since the diagnosis of diffuse large B-cell lymphoma. Unfortunately, the therapeutic result was not good. Other immuno-chemotherapy regimen RICE (rituximab, ifosfamide, carboplatin, etoposide) began to be implemented without success. The abdominal mass still increased, and anemia persisted. Thus, the need of blood transfusion was increased. The first time to detect the ABO blood group was on November 7, 2020, and his ABO blood group was initially typed as B. He was transfused the B type leukocyte poor RBCs (LPR) was on November 19, 2020 for the first time. The efficiency of blood transfusion seemed not be good (Figure 1). It was surprised that ABO blood group discrepancy of the patient was found on December 2, 2020 by forward and reverse typing methods (Table 1). Thus, the patient was referred for ABO genotyping and he began to transfuse O type washed RBCs (WRBC) on December 3, 2020. Blood samples from family members of the proband were also obtained and analyzed for the pedigree investigation. Compared to transfuse B type leukocyte poor RBCs (LPR), the efficiency of transfusing O type washed RBCs (WRBC) was better. Although hemoglobin level did not greatly improve, indirect bilirubin level evidently decreased (Figure 1). Furthermore, the patient told us his anemia symptoms had improved a lot when he was transfused O type washed RBCs (WRBC). It was worthy of noting that we could not eliminate interference of disease progression and therapy on transfusion efficiency comparison, and we could not guarantee the standardization of transfusion values. But we believed O type WRBC transfusion was safer for the patient.

**TABLE 1:** Serological results of the proband at different temperatures.

Temperature	Forward typing					Reverse typing				
	Anti-A	Anti-B	Anti-D	Anti-A,B	Anti-H	Ac	Bc	Oc	A1c	Auto
4°C	0	4+	4+	/	/	1+	2+	0	1+	0
RT	0	4+	4+	/	4+	2+	1+	0	w	0
37°C	0	4+	4+	/	/	1+	1+	0	w	0

*W, weak reaction; c, cells; /, no detect. The agglutination score ranges from 0 to 4+.*

The RBCs of the proband were strongly agglutinated with 4 + reaction with anti-B and anti-H at room temperature (RT) in the forward typing, and the RBCs of the proband had no agglutination with anti-A. But the serum of the proband demonstrated 2 + agglutination with A cells, weak agglutination with A1 cells, 1 + agglutination with B cells at RT in the reverse typing. No evidence of reactivity was found between the patient’s RBCs and his serum at RT. Furthermore, no evidence of reactivity was also found between O cells and the proband’s serum at RT (Table 1). The results of the forward and reverse typing at 4°C and 37°C were also showed in Table 1. And it was strange that the serum of the proband agglutinated both A and B cells in reverse typing test. The cell group was confirmed by the use of grouping sera from several sources, which indicated that the discrepancy was not due to an irregular erythrocyte antigen but to the presence of an atypical agglutinin in the serum.

No evidence of reactivity was also found between O cells and the proband’s serum at 4°C (Table 1). Therefore, the interference of specific cold agglutinins on determining ABO blood group could be excluded. From the results, we could determine the agglutination of the proband’s serum and B cells was true, but we did not exactly know the specific antibody was anti-A,B or anti-B. Supplementary Table 1 showed the serum was only detected 2 + agglutination with standard B cells but not standard human A cells at RT for 15 min in the absorption-elution test. Thus, the specific antibody was anti-B. Furthermore, dithiothreitol treatment abolished all agglutinin activity of the serum, suggesting that the antibody was IgM, not IgG (data non-shown). However, the mechanism through which the IgM anti-B antibody was produced was still unclear.

**FIGURE 2:**
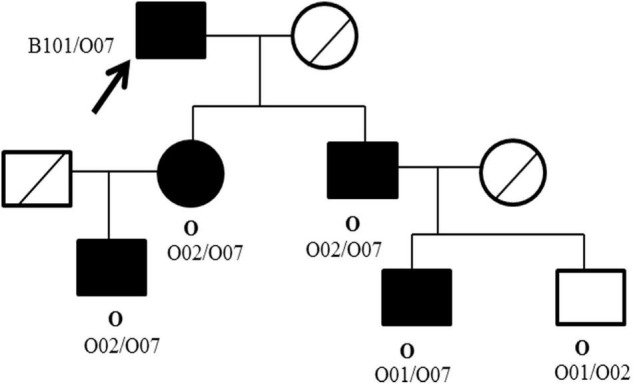
Pedigree of the ABO phenotype and genotype of the proband and family members. The ABO phenotype is in bold (above), whereas the genotype is in plain text (below). □ Male; ○ Female; ●■ Carriers with O07 allele; ╱ Samples not acquired; ↗ The proband.

**FIGURE 3:**
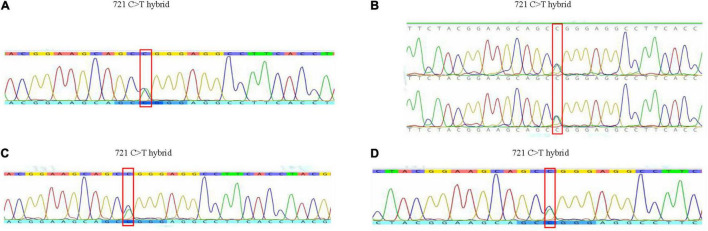
The results of gene sequence of the family in exon 6 and 7. **(A)** Gene sequence of the proband. **(B)** Gene sequence of his daughter and son. **(C)** Gene sequence of his grandson 1. **(D)** Gene sequence of his grandson 3.

Pedigree blood investigation showed the proband’s son, daughter and three grandsons were all typical O phenotype (Figure 2). Compared with the published O consensus sequence (O101), the proband’s sample was heterozygous for an A > G exchange at position 297, a T > A exchange at position 646, a G > A exchange at position 681, a C > T exchange at position 721, a C > T exchange at position 771 and a G > A at position 829. These nucleotide exchanges resulted in a Thr to Pro substitution at codon 88 in the ABO protein. The ABO genotypes of the proband and his family members were listed in Supplementary Table 2. Exon 6 and 7 cloning sequencing confirmed that c.721 C > T was associated with an O07 allele (Figure 3). O07 maybe play a vital role in B-cell lymphoma affecting the proband’s ABO blood group.

## Discussion

In this report, ABO blood typing results of the proband showed discrepancy between forward and reverse typing. The causes of discrepancy are usually classified into two categories: technical discrepancy and clinical discrepancy. Technical discrepancy includes the following: mixing of blood samples; non-calibrated centrifuges; clerical errors; cells suspension either too light or too heavy; missed identification of blood specimen (11). Technical errors could be excluded after our repeated confirmation in this study. Clinical discrepancies are classified into the following types: Group I discrepancy: weak reacting or missing antibodies; Group II discrepancy: weak reacting or missing antigens; Group III discrepancy: protein/plasma abnormality leading to rouleaux formation; Group IV discrepancy: miscellaneous problems antibodies (11). In this report, the cause of discrepancy may be Group IV.

The patient’s ABO blood group was initially typed as B on November 7, 2020, but it was very strange that serum of the proband agglutinated both A and B cells in reverse typing on December 2, 2020. To resolve the ABO discrepancy, we performed a series of tests described above. Autoantibodies against red blood cells (RBCs) optimally but not exclusively reacting in the cold (0°C) are termed cold agglutinins (CAs). To the best of our knowledge, common cold agglutinins were multispecific antibodies targeted to carbohydrate antigen. CAs aimed at the 3 major groups of Ii, Pr/Sa, and Sia-l1, -b1, -lb1,2 antigens have been identified (12). No evidence of reactivity was also found between O cells and the proband’s serum at 4°C, RT and 37°C in this case (Table 1). Accordingly, we could determine the agglutination of the proband’s serum and B cells was true, but we did not exactly know the specific antibody was anti-A,B or anti-B. Absorption-elution test results showed that the specific antibody was anti-B. Furthermore, dithiothreitol treatment test results suggested that the antibody was IgM, not IgG. Compared to transfuse B type leukocyte poor RBCs (LPR), the efficiency of transfusing O type washed RBCs (WRBC) was better. It was worthy of noting that we could not eliminate interference of disease progression and therapy on transfusion efficiency comparison, and we could not guarantee the standardization of transfusion values. But we believed O type WRBC transfusion was safer for the patient.

Pedigree blood investigation showed the proband’s son, daughter and grandsons were all typical O phenotype. Direct DNA sequencing results showed the proband possessed the O07 allele. His son, daughter and his three grandsons were also found to possess the O07 allele. O07 was a rare allele, which was first identified in 2009 in Chinese Han population (13). The serological results of O07 have not been reported so far. New allele could influence glycosyltransferase activity, thus interfere the ABO antigen expressions (14–16), and further influenced the production of antibodies. For this reason, we speculated O07 allele maybe play a vital role in B-cell lymphoma affecting blood transfusion strategy, but no evidences existed so far. It was worth noting that B-cell lymphoma therapy and ABO gene methylation maybe produce the anti-B antibody, but this assumption could not be proved in this report.

NHLs are a heterogeneous group of malignancies derived from the immune system cells. About 85–90% of NHLs are originated from B cells, whereas the remaining lymphomas are derived from T cells or NK cells (17). Diffuse large B-cell lymphoma (DLBCL) is the most common and aggressive type of B-cell lymphoma, accounting for 30–35% of non-Hodgkin lymphomas (18). Owing to the advances of molecular medicine in the past years, opportunities for targeted therapy with novel molecules were revealed, and an improved survival in patients with DLBCL was observed (19). The advantages of immuno-chemotherapy have been verified in a whole series of lymphoma subtypes. Rituximab emerged as the first monoclonal (mAb) antibody to target the CD20 molecule (anti-CD20) have been the standard of care in the induction treatment of DLBCL (20). Furthermore, the therapeutic effect of R-CHOP treatment regimen was better than CHOP (21). Nevertheless, the therapeutic effect of R-CHOP treatment regimen was not good in this report, it maybe rituximab resistance.

As we well known, the immune system of non-Hodgkin lymphomas is abnormal, most B-cell and T-cell NHLs show clonal rearrangement of their immunoglobulin (IG) and T-cell receptor (TCR) genes, respectively (22). Furthermore, a study showed that lymphoma could produce anti-B1 cold agglutinin, resulting in discrepancy of forward and reverse typing (9). In this report, B-cell lymphoma maybe produce IgM anti-B antibody and could interfere the blood transfusion strategy, which suggested clinicians to be careful when the patients were diagnosed with B-cell lymphoma.

## Ethics Statement

The studies involving human participants were reviewed and approved by the Ethics Committee of Zhejiang University. The patients/participants provided their written informed consent to participate in this study.

## Author Contributions

ZL conceived and designed the study. FJ collected the clinical data and drafted the manuscript. TS and YW participated in the serological study and analysis of the data. FJ, TS, YW, and ZL were responsible for revising this manuscript. All authors contributed to the article and approved the submitted version.

## Conflict of Interest

The authors declare that the research was conducted in the absence of any commercial or financial relationships that could be construed as a potential conflict of interest.

## Publisher’s Note

All claims expressed in this article are solely those of the authors and do not necessarily represent those of their affiliated organizations, or those of the publisher, the editors and the reviewers. Any product that may be evaluated in this article, or claim that may be made by its manufacturer, is not guaranteed or endorsed by the publisher.

## References

[1] Liumbruno GM Franchini M. Beyond immunohaematology: the role of the ABO blood group in human diseases. *Blood Transfus.* (2013) 11:491–9. 10.2450/2013.0152-13 24120598 PMC3827391

[2] Franchini M Favaloro EJ Targher G Lippi G. ABO blood group, hypercoagulability, and cardiovascular and cancer risk. *Crit Rev Clin Lab Sci.* (2012) 49:137–49. 10.3109/10408363.2012.708647 22856614

[3] Franchini M Liumbruno GM. ABO blood group: old dogma, new perspectives. *Clin Chem Lab Med.* (2013) 51:1545–53. 10.1515/cclm-2013-0168 23648637

[4] Zhang H Mooney CJ Reilly MP. ABO blood groups and cardiovascular diseases. *Int J Vasc Med.* (2012) 2012:641917. 10.1155/2012/641917 PMC3485501 23133757

[5] Swerdlow SH Campo E Pileri SA Harris NL Stein H Siebert R The 2016 revision of the World Health Organization classification of lymphoid neoplasms. *Blood.* (2016) 127:2375–90. 26980727 10.1182/blood-2016-01-643569 PMC4874220

[6] Perry AM Diebold J Nathwani BN MacLennan KA Müller-Hermelink HK Bast M Non-Hodgkin lymphoma in the developing world: review of 4539 cases from the International Non-Hodgkin lymphoma classification project. *Haematologica.* (2016) 101:1244–50. 10.3324/haematol.2016.148809 27354024 PMC5046654

[7] Teras LR DeSantis CE Cerhan JR Morton LM Jemal A Flowers CR. 2016 US lymphoid malignancy statistics by World Health Organization subtypes. *CA Cancer J Clin.* (2016) 66:443–59. 10.3322/caac.21357 27618563

[8] Loddenkemper C Anagnostopoulos I Hummel M Jöhrens-Leder K Foss HD Jundt F Differential emu enhancer activity and expression of BOB.1/OBF.1, Oct2, PU.1, and immunoglobulin in reactive B-cell populations, B-cell non-Hodgkin lymphomas, and Hodgkin lymphomas. *J Pathol.* (2004) 202:60–9. 10.1002/path.1485 14694522

[9] Dodds AJ Klarkowski D Cooper DA Isbister JP. In-vitro synthesis of an anti-BI cold agglutinin complicating a case of lymphoma. *Am J Clin Pathol.* (1979) 71:473–5. 10.1093/ajcp/71.4.473 443208

[10] Riley DS Barber MS Kienle GS Aronson JK von Schoen-Angerer T Tugwell P CARE guidelines for case reports: explanation and elaboration document. *J Clin Epidemiol.* (2017) 89:218–35. 10.1016/j.jclinepi.2017.04.026 28529185

[11] Harmening D. *Modern Blood Banking and Transfusion Practice.* New Delhi: Jaypee Publishers (2013). p. 136–43.

[12] Roelcke D König AL Seyfert UT Pereira A. Coexisting anti-I/i plus anti Pr cold agglutinins in individual sera. *Infusionsther Transfusionsmed.* (2000) 27:149–53. 10.1159/000025260 10878484

[13] Zhu F Tao S Xu X Ying Y Hong X Zhu H Distribution of ABO blood group allele and identification of three novel alleles in the Chinese Han population. *Vox Sang.* (2010) 98:554–9. 10.1111/j.1423-0410.2009.01291.x 20003128

[14] Patnaik SK Helmberg W Blumenfeld OO. BGMUT: NCBI dbRBC database of allelic variations of genes encoding antigens of blood group systems. *Nucleic Acids Res.* (2012) 40:D1023–9. 10.1093/nar/gkr958 22084196 PMC3245102

[15] Seltsam A Hallensleben M Kollmann A Blasczyk R. The nature of diversity and diversification at the ABO locus. *Blood.* (2003) 102:3035–42. 10.1182/blood-2003-03-0955 12829588

[16] Seltsam A Hallensleben M Kollmann A Burkhart J Blasczyk R. Systematic analysis of the ABO gene diversity within exons 6 and 7 by PCR screening reveals new ABO alleles. *Transfusion.* (2003) 43:428–39. 10.1046/j.1537-2995.2003.00321.x 12662274

[17] Sabattini E Bacci F Sagramoso C Pileri SA. WHO classification of tumours of haematopoietic and lymphoid tissues in 2008: an overview. *Pathologica.* (2010) 102:83–7. 21171509

[18] Menon MP Pittaluga S Jaffe ES. The histological and biological spectrum of diffuse large B-cell lymphoma in the World Health Organization classification. *Cancer J.* (2012) 18:411–20. 10.1097/PPO.0b013e31826aee97 23006945 PMC3458515

[19] Eichenauer DA Engert A Schulz H. Expanded use of rituximab in the management of non-Hodgkin lymphoma. *Onco Targets Ther.* (2009) 2:189–97. 10.2147/ott.s3322 20616906 PMC2886316

[20] Solimando AG Ribatti D Vacca A Einsele H. Targeting B-cell non Hodgkin lymphoma: new and old tricks. *Leuk Res.* (2016) 42:93–104. 10.1016/j.leukres.2015.11.001 26818572

[21] Coiffier B Thieblemont C Van Den Neste E Lepeu G Plantier I Castaigne S Long-term outcome of patients in the LNH-98.5 trial, the first randomized study comparing rituximab-CHOP to standard CHOP chemotherapy in DLBCL patients: a study by the groupe d’etudes des lymphomes de l’adulte. *Blood.* (2010) 116:2040–5. 10.1182/blood-2010-03-276246 20548096 PMC2951853

[22] van Dongen JJ Langerak AW Brüggemann M Evans PA Hummel M Lavender FL Design and standardization of PCR primers and protocols for detection of clonal immunoglobulin and T-cell receptor gene recombinations in suspect lymphoproliferations: report of the BIOMED-2 Concerted Action BMH4-CT98-3936. *Leukemia.* (2003) 17:2257–317. 10.1038/sj.leu.2403202 14671650

