# Supplementary material for: B-Cell Lymphoma Producing IgM Anti-B Antibody: A Case Report
Source: Front Med (Lausanne). 2022 May 16;9:904296. doi: 10.3389/fmed.2022.904296 (PMC9150775; doi:10.3389/fmed.2022.904296)
Supplement: Supplementary file 2 [file Table_1.docx]

**SUPPLEMENTARY TABLE 1** Absorption-elution test results of the proband.

|  |  | | Ac | Bc | Oc |
| --- | --- | --- | --- | --- | --- |
| Control | |  | 1+ | 4+ | 0 |
| A cell absorption-elution | | Immediately centrifuge | w+ | 0 | 0 |
|  |  | RT for 15 min | 1+ | 0 | 0 |
| B cell absorption-elution | | Immediately centrifuge | 0 | 1+s | 0 |
|  |  | RT for 15 min | 0 | 2+ | 0 |

*S, agglutination intensity was stronger; w, weak reaction; c, cells. The agglutination score ranges from 0 to 4+.*

**SUPPLEMENTARY TABLE 2** Partial polymorphism of the ABO gene in the proband and his family members.

| Position | 261 | 297 | 467 | 526 | 646 | 657 | 681 | 703 | 721 | 771 | 796 | 803 | 829 | 930 | Genotype |
| --- | --- | --- | --- | --- | --- | --- | --- | --- | --- | --- | --- | --- | --- | --- | --- |
| Reference | G | A | C | C | T | C | G | G | C | C | C | G | G | G | A101 |
| Reference | G | G | C | G | T | T | G | A | C | C | A | C | G | A | B101 |
| Reference | del | A | C | C | T | C | G | G | C | C | C | G | G | G | O101 |
| Proband | del/G | G/G | C/C | C/G | A/T | C/T | A/G | A/G | C/T | C/T | A/C | C/G | A/G | A/G | B101/O07 |
| Son | del | G/G | C/C | C/C | A/A | C/C | A/A | G/G | C/T | T/T | C/C | G/G | A/A | G/G | O02/O07 |
| Daughter | del | G/G | C/C | C/C | A/A | C/C | A/A | G/G | C/T | T/T | C/C | G/G | A/A | G/G | O02/O07 |
| Grandson 1 | del/del | A/G | C/C | C/C | A/T | C/C | A/G | G/G | C/T | C/T | C/C | G/G | A/G | G/G | O01/O07 |
| Grandson 2 | del/del | A/G | C/C | C/C | A/T | C/C | A/G | G/G | / | C/T | C/C | G/G | A/G | G/G | O01/O02 |
| Grandson 3 | del/del | G/G | C/C | C/C | A/A | C/C | A/A | G/G | C/T | T/T | C/C | G/G | A/A | G/G | O02/O07 |

*/, no detect.*
